# A Mesalazine–Aloperine Co-Amorphous System for Simultaneous Dissolution Enhancement, Permeability Improvement, and Synergistic Anti-Ulcerative Colitis Activity: A Combined Pharmaceutics and Network Pharmacology Study

**DOI:** 10.3390/pharmaceutics18101288

**Published:** 2026-10-10

**Authors:** Huan Su, Ningzi Guo, Song Yuan, Junyi Huang, Ningyi Wei, Hui Xu, Yang Liu

**Affiliations:** 1 Key Laboratory of Molecular Pharmacology and Drug Evaluation, School of Pharmacy, Ministry of Education, Yantai University, Yantai 264005, China; 18856527356@163.com (H.S.); guoningzi@nidc.org.cn (N.G.); 2 Institute for Chemical Drug Control, National Institutes for Drug Control, No. 31, Huatuo Road, Beijing 102629, China; yuansong@nidc.org.cn (S.Y.); hjy123tt@163.com (J.H.); weiny@nidc.org.cn (N.W.)

**Keywords:** mesalazine, aloperine, co-amorphous system, ulcerative colitis, dissolution, in vitro permeability, network pharmacology

## Abstract

**Background/Objectives:** Mesalazine (MSZ), the first-line therapy for ulcerative colitis (UC), is a BCS Class IV drug limited by poor aqueous solubility and low intestinal permeability, which together restrict colonic drug delivery and therapeutic efficacy. This study aimed to develop a co-amorphous (CAM) system of MSZ and aloperine (ALO) to enhance MSZ dissolution and permeation while achieving anti-UC synergy. **Methods:** The MSZ-ALO CAM system was prepared via solvent evaporation and characterized by PXRD, DSC, FTIR, and ^1^H NMR. Equilibrium solubility, intrinsic dissolution rate (IDR), and in vitro permeability were assessed under biorelevant pH conditions. Physical stability was evaluated under long-term and accelerated conditions. Network pharmacology predicted potential therapeutic targets and synergistic mechanisms. **Results:** Solid-state characterization confirmed a homogeneous single-phase CAM system stabilized mainly by a carboxylate–ammonium ionic bond and hydrogen bonding. At pH 4.5, where MSZ solubility was lowest, equilibrium solubility and IDR increased up to 71.9-fold and 35.8-fold, respectively. In vitro permeability at pH 6.5 increased 2-fold. The system remained physically stable for eight months under dry conditions and withstood accelerated stresses without recrystallization. Network pharmacology identified 51 shared targets among MSZ, ALO, and UC, enriched in MAPK, HIF-1, and AMPK pathways; core hubs included HSP90AA1, SRC, CASP3, GSK3B, and ALB. **Conclusions:** This study presented the first MSZ-ALO CAM system that simultaneously addresses the dual solubility and permeability barriers of MSZ and provided a multi-target pharmacological basis for synergistic UC therapy, offering a promising pharmaceutical strategy for colon-targeted delivery.

## 1. Introduction

Ulcerative colitis (UC) is a chronic, relapsing, immune-mediated inflammatory disease of the colonic mucosa that imposes a substantial and growing global health burden [1]. Mesalazine (MSZ) is a first-line agent for inducing and maintaining remission in mild-to-moderate UC, acting primarily through local anti-inflammatory effects on the colonic mucosa [2]. However, MSZ is a BCS Class IV drug with both poor aqueous solubility (only 1.41 mg/mL in water) and low intestinal permeability, which is readily absorbed and metabolized in the upper gastrointestinal tract, leading to insufficient drug levels in the inflamed colon along with a relatively low oral bioavailability of about 28% [3,4]. To maintain effective therapeutic concentrations, high and frequent dosing is required in clinical practice, which not only increases the medication burden on patients but also predisposes them to adverse effects such as gastrointestinal discomfort and hepato-renal toxicity [5]. Since UC primarily affects the colonic mucosa, local drug delivery represents an especially suitable therapeutic strategy. Rectal MSZ suppositories and enemas are first-line agents endorsed by clinical guidelines for distal UC and proctitis. These formulations achieve high drug concentrations within the colonic lumen and inflamed mucosa at target lesions, while limiting systemic drug exposure [6]. Nonetheless, currently available topical MSZ preparations are hampered by several drawbacks such as restricted drug dissolution at inflamed sites, erratic proximal spreading of formulations, inadequate mucosal retention [7], and poor patient adherence largely attributable to the large required enema volume [8]. Various formulation strategies have been explored to improve the solubility and bioavailability of MSZ. Among them, co-crystals represent a promising approach that can modulate the solubility and dissolution behavior of poorly water-soluble drugs without altering their chemical structure [9]. Several MSZ co-crystals have been reported. For example, an MSZ-ascorbic acid co-crystal (1:2 molar ratio) exhibited a 7-fold increase in solubility in distilled water and significantly superior drug release compared with a commercial formulation [3], and an MSZ-glutamine co-crystal was developed to optimize physicochemical properties for colonic targeted delivery [10]. Nevertheless, crystallinity imposes inherent limitations that are difficult to circumvent: the thermodynamic stability of the crystal lattice sets an upper limit on the degree of solubility enhancement [11,12], and such systems are susceptible to physical-instability problems, including dissociation and phase separation, during processing and storage [13,14].

Co-amorphous (CAM) systems have emerged in recent years as a novel solid-state modification technology. They are formed by combining two or more low-molecular-weight components into a homogeneous single-phase amorphous system through various intermolecular interactions, such as hydrogen bonding, π–π stacking, and even ionic interactions [15,16,17]. CAM systems can substantially enhance the solubility and dissolution rate of poorly water-soluble drugs by exploiting the high-energy state of the amorphous form while simultaneously improving physical stability through intermolecular hydrogen bonds and ionic interactions [18,19]. Drug–drug CAM systems composed of two active pharmaceutical ingredients can further enable co-delivery and synergistic therapeutic efficacy while improving dissolution and stability [15,20,21], and thus have become a focus of pharmaceutical research. Notably, in terms of potential local colonic delivery, the high-energy amorphous nature of CAM facilitates rapid dissolution of both components within the limited volume of colonic fluid, thereby elevating the concentration of dissolved MSZ available for local therapeutic action. The stabilized intermolecular interactions can suppress moisture-triggered recrystallization. Given that the local therapeutic efficacy of MSZ is governed by the free drug concentration at the mucosal surface instead of the administered total dose [22], improved solubility may, in principle, lower the MSZ dose required to attain equivalent therapeutic effects. However, to the best of our knowledge, no co-amorphous drug–drug system incorporating MSZ has yet been reported.

**Figure 1:**
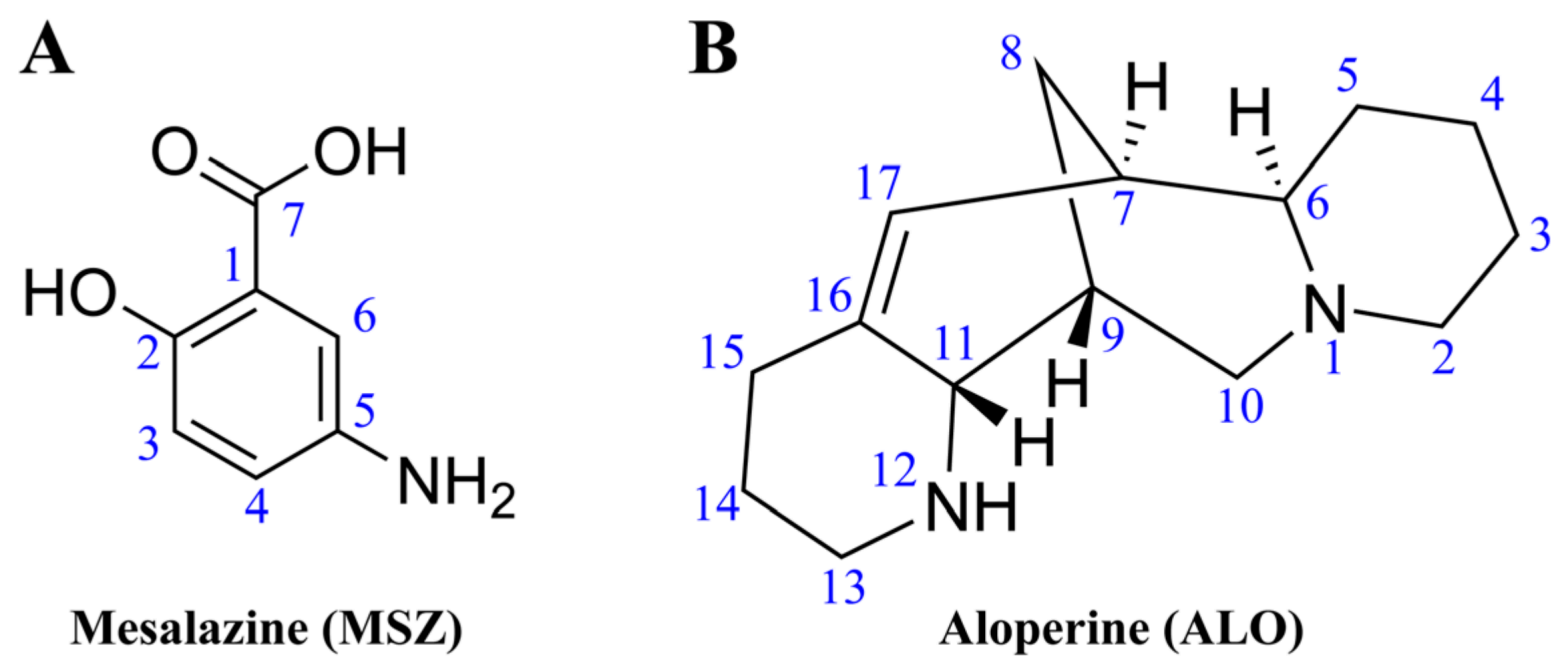
Chemical structures of Mesalazine (**A**) and Aloperine (**B**).

Aloperine (ALO) is a quinolizidine alkaloid extracted from the leguminous plant *Sophora alopecuroides* L. and possesses significant anti-inflammatory, antioxidant, and immunomodulatory activities [23]. ALO has been employed as a common co-former in CAM systems, such as the CAM systems of febuxostat–aloperine [24] and wogonin–aloperine [25]. Moreover, accumulating studies have verified that ALO confers marked therapeutic effects in experimental colitis: it mitigates colonic inflammation and mucosal damage, and decreases the disease activity index [23,26,27]. These findings imply that ALO may exert synergistic therapeutic effects with MSZ. Nevertheless, oral administration of ALO at higher doses elicits safety concerns related to systemic drug exposure and tolerability [28]. Accordingly, local delivery of ALO to the colonic mucosa represents an appealing strategy, as it can sustain high drug concentrations in inflamed tissues while lowering the total administered dose. While this local delivery modality was not assessed in the current investigation, such a therapeutic rationale underpins the design of the CAM powder as a proof-of-concept solid-state system and motivated its development. As shown in Figure 1, the chemical structures of MSZ and ALO allow the formation of an ionic bond via acid–base neutralization and the establishment of an extensive hydrogen-bonding network through hydroxyl and carbonyl groups. These strong intermolecular interactions provide a structural basis for constructing a stable CAM system [29].

Herein, an MSZ-ALO CAM system was developed, representing, to the best of our knowledge, the first drug–drug CAM system of MSZ to incorporate a small-molecule natural product from traditional Chinese medicine. Its formation was verified by solid-state characterization, and the solubility, dissolution rate, permeability, and physical stability of the system were investigated to evaluate the potential of the CAM to enhance the solubility and permeability of MSZ. Furthermore, network pharmacology was employed to explore the potential anti-UC mechanisms of the combination, with the aim of achieving the dual advantages of pharmaceutical solubilization enhancement and pharmacological synergy. The present work focused on the CAM powder and provided a basis for the subsequent development of formulations for localized administration.

## 2. Materials and Methods

### 2.1. Materials

Mesalazine (MSZ, purity ≥ 99%) was purchased from Shanghai Aladdin Biochemical Technology Co., Ltd. (Shanghai, China). Aloperine (ALO, purity = 97%) was obtained from Shanghai Macklin Biochemical Co., Ltd. (Shanghai, China). HPLC-grade methanol and ethanol (purity > 99.9%) were sourced from Thermo Fisher Scientific (Waltham, MA, USA). Dipotassium hydrogen phosphate and phosphoric acid were purchased from Sinopharm Chemical Reagent Co., Ltd. (Shanghai, China), and 40% tetrabutylammonium hydroxide aqueous solution was purchased from Shanghai Aladdin Biochemical Technology Co., Ltd. (Shanghai, China).

### 2.2. Preparation of Co-Amorphous System

#### 2.2.1. Preparation of Co-Amorphous Samples by Solvent Evaporation

MSZ (15.31 mg) and ALO (23.23 mg) were weighed into a vial, and 2 mL of 95% ethanol was added. The mixture was stirred with a magnetic stirrer at 600 rpm at room temperature for 8 h, yielding a clear and transparent solution. The solution was then transferred to a vacuum drying oven and dried under vacuum at 40 °C for 2–4 h. After complete evaporation of the solvent, the MSZ-ALO CAM (1:1) was obtained.

#### 2.2.2. Preparation of Physical Mixture

MSZ (15.31 mg) and ALO (23.23 mg) were weighed into a mortar and manually mixed for 1 min to yield the MSZ-ALO physical mixture, designated as PM.

### 2.3. Solid-State Characterization

#### 2.3.1. Powder X-Ray Diffraction (PXRD)

PXRD patterns were collected using an X-ray powder diffractometer (Rigaku, Tokyo, Japan) with Cu Kα1 radiation at 20 kV and 10 mA. The scanning range of 2*θ* was set from 3° to 40°, while samples for stability evaluation were scanned up to 80°. To evaluate the long-term physical stability of the MSZ-ALO CAM, samples were stored in a desiccator at 25 °C for eight months. In the accelerated stability study [30], samples were exposed to a high temperature of 50 °C, to high humidity (75 ± 5% relative humidity, 25 °C), and to light (4500 ± 500 lx) for ten days. All samples were monitored by PXRD.

#### 2.3.2. Differential Scanning Calorimetry (DSC)

DSC measurements were performed on a METTLER DSC 3 (Mettler Toledo, Zurich, Switzerland). Approximately 3 mg of each sample was sealed in an aluminum crucible with a pierced lid, and heated under a nitrogen flow of 20 mL/min at a heating rate of 10 °C/min. The temperature ranges were 30–300 °C for MSZ, 30–110 °C for ALO, and 0–120 °C for MSZ-ALO CAM. The glass transition temperature was determined from the midpoint of the step change in the heat flow curve.

#### 2.3.3. Fourier Transform Infrared Spectroscopy (FTIR)

FTIR spectra were recorded on a PerkinElmer 400 infrared spectrometer (PerkinElmer, Waltham, MA, USA) using the potassium bromide (KBr) pellet method. Spectra were acquired over the range of 400–4000 cm^−1^ for MSZ, ALO, MSZ-ALO CAM, and PM.

#### 2.3.4. Nuclear Magnetic Resonance (NMR)

^1^H NMR spectra of MSZ, ALO, and the MSZ-ALO CAM system were recorded on a Bruker AVANCE 600 MHz spectrometer (Billerica, MA, USA) using deuterated dimethyl sulfoxide (DMSO-d_6_) as the solvent.

### 2.4. Solubility and Dissolution Rate Studies

#### 2.4.1. Equilibrium Solubility Determination

The equilibrium solubility was determined in buffer systems at pH 1.2, pH 4.5, and pH 6.8 at 37.0 ± 0.5 °C, using crystalline MSZ, MSZ-ALO CAM, or PM as the solid phase, respectively. An excess of each sample was added to 3 mL of the respective buffer solution and shaken in a thermostatic shaker at 150 rpm for 24 h. After dilution, samples were analyzed using a Shimadzu LC-30A high-performance liquid chromatograph (Shimadzu Corporation, Kyoto, Japan). The chromatographic conditions were as follows: a Waters XTERRA^®^ MS C18 column (3.9 × 150 mm, 5 μm) maintained at 30 °C; a mobile phase composed of a phosphate buffer (4.35 g of dipotassium hydrogen phosphate dissolved in 1000 mL of water, adjusted to pH 7.2 with phosphoric acid, and then mixed with 3.0 mL of 40% tetrabutylammonium hydroxide aqueous solution) and methanol (72:28, *v*/*v*); a flow rate of 1 mL/min; and UV detection at 240 nm. Quantification was performed by the external standard method, and each experiment was repeated three times.

#### 2.4.2. Intrinsic Dissolution Rate (IDR) Determination

Approximately 10 mg of MSZ, MSZ-ALO CAM, and PM was placed into an intrinsic dissolution apparatus (Pion Inc., Billerica, MA, USA) and compressed at 150 psi for 1.5 min to form pellets with a fixed exposed surface area of 0.0707 cm^2^. The pellets were immersed in 15 mL of pH 6.8, pH 4.5, or pH 1.2 buffer medium at 37 ± 0.5 °C and stirred at 150 rpm. Dissolved MSZ was monitored every 30 s for 10 min using a 2 mm fiber-optic probe. Each experiment was repeated three times. The IDR was calculated based on the first six minutes using the following formula [31]:

(1) $$IDR=\frac{dc}{dt}\times \frac{V}{A}$$

where IDR [μg/(min·cm^2^)] is the intrinsic dissolution rate, *V* (mL) is the volume of the dissolution medium, d*c*/d*t* [μg/(min·mL)] is the linear slope of the dissolution curve, and *A* (cm^2^) is the exposed surface area of the pellet.

### 2.5. Permeability Measurement

Permeability was assessed using a Pion µFlux biomimetic permeation analyzer (Pion Inc., Billerica, MA, USA). A membrane with an effective surface area of 1.65 cm^2^ was coated with biomimetic intestinal lipids to simulate the intestinal barrier. The donor compartments were filled with pH 6.5 and pH 5.0 intestinal buffers, respectively. The acceptor compartment contained 20 mL of surfactant-containing ASB buffer (to maintain sink conditions) and was maintained at 37 ± 0.5 °C with stirring at 200 rpm. Stock solutions of MSZ, MSZ-ALO CAM, and PM were separately prepared in DMSO and introduced into the donor compartment. MSZ in the acceptor compartment was monitored in real time using a fiber-optic UV probe over 14.5 h at 5 min intervals. The permeation flux (*J*) and effective permeability (*P*_e_) were calculated as follows [32]:

(2) $$J=\frac{dc}{dt}\times \frac{V}{A}$$

(3) $$P_{e}=\frac{J}{C_{t}\cdot 60}$$

where *J* [µg/(min·cm^2^)] is the permeation flux, defined as the total mass transported across a unit membrane area per unit time; d*c*/d*t* [µg/(mL·min)] is the slope of the concentration–time curve in the acceptor compartment; *V* (mL) is the acceptor compartment volume; A (cm^2^) is the effective membrane surface area; *P*_e_ (cm/s) is the effective permeability; and *C_t_* (µg/mL) is the average drug concentration in the donor compartment.

### 2.6. Network Pharmacology Analysis

The potential target candidates of MSZ and ALO were predicted using the PharmMapper (https://www.lilab-ecust.cn/pharmmapper/ (accessed on 3 October 2026)) and SwissTargetPrediction (https://swisstargetprediction.ch (accessed on 3 October 2026)) databases. The two-dimensional structures of MSZ and ALO were uploaded to retrieve potential targets, which were limited to human proteins. Protein names were standardized via the UniProt database (https://www.uniprot.org/ (accessed on 3 October 2026)). UC-related genes were obtained from the OMIM (https://www.omim.org/ (accessed on 3 October 2026)) and GeneCards (https://www.genecards.org/ (accessed on 3 October 2026)) databases using “Ulcerative Colitis” as the search term. A Venn diagram was constructed using the Jvenn tool (https://jvenn.toulouse.inra.fr/ (accessed on 3 October 2026)) to identify the common targets shared between the drug combination and UC. The intersecting targets were imported into the STRING database (https://string-db.org/ (accessed on 3 October 2026)) to build a protein–protein interaction (PPI) network, which was subsequently visualized and analyzed with Cytoscape 3.10.3. Gene Ontology (GO) enrichment analysis (covering biological processes, BP; cellular components, CC; and molecular functions, MF) and Kyoto Encyclopedia of Genes and Genomes (KEGG) pathway enrichment were performed using Metascape (https://metascape.org/ (accessed on 3 October 2026)). GO enrichment plots were generated using the bioinformatics cloud platform (http://www.bioinformatics.com.cn (accessed on 3 October 2026)).

## 3. Results and Discussion

### 3.1. PXRD Verification of Co-Amorphous Formation

**Figure 2:**
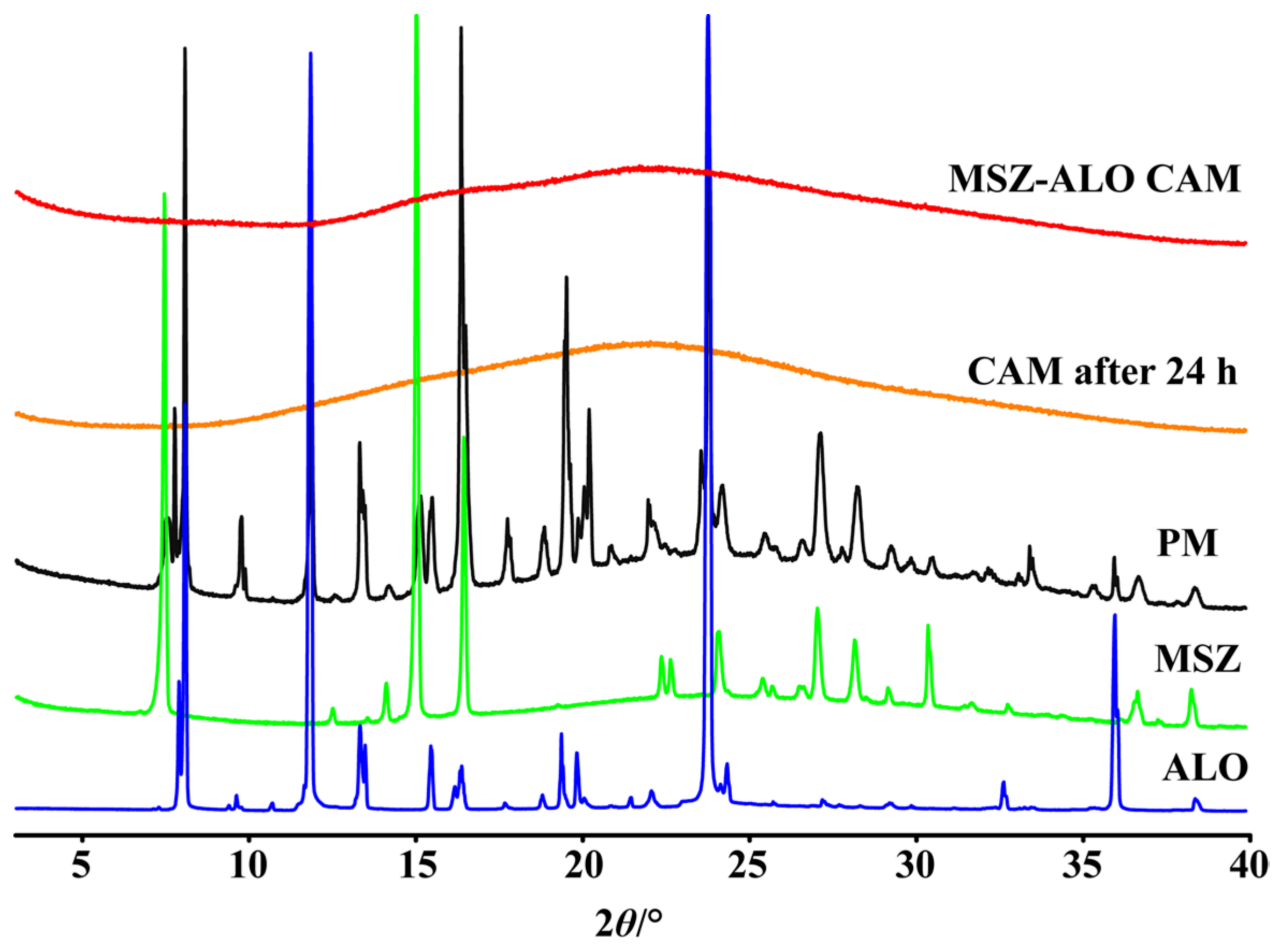
PXRD patterns of MSZ, ALO, PM, and MSZ-ALO CAM before and after 24 h equilibrium solubility.

The MSZ-ALO CAM system was successfully prepared via solvent evaporation, and PXRD verified its formation, as shown in Figure 2. The PXRD pattern of MSZ displayed characteristic diffraction peaks at 2*θ* = 7.46°, 15.05°, 16.47°, 26.56°, 27.11°, and 30.44°, while ALO exhibited characteristic peaks at 2*θ* = 8.08°, 9.63°, 11.86°, 13.50°, 16.41°, and 23.83°, consistent with the literature [24,33,34]. The PM merely showed a simple superposition of the diffraction peaks of the two parent drugs without the formation of any new phase. The MSZ-ALO CAM exhibited only a single broad halo pattern devoid of any sharp crystalline diffraction peaks, confirming the successful conversion to the amorphous state [35].

### 3.2. DSC Analysis

**Figure 3:**
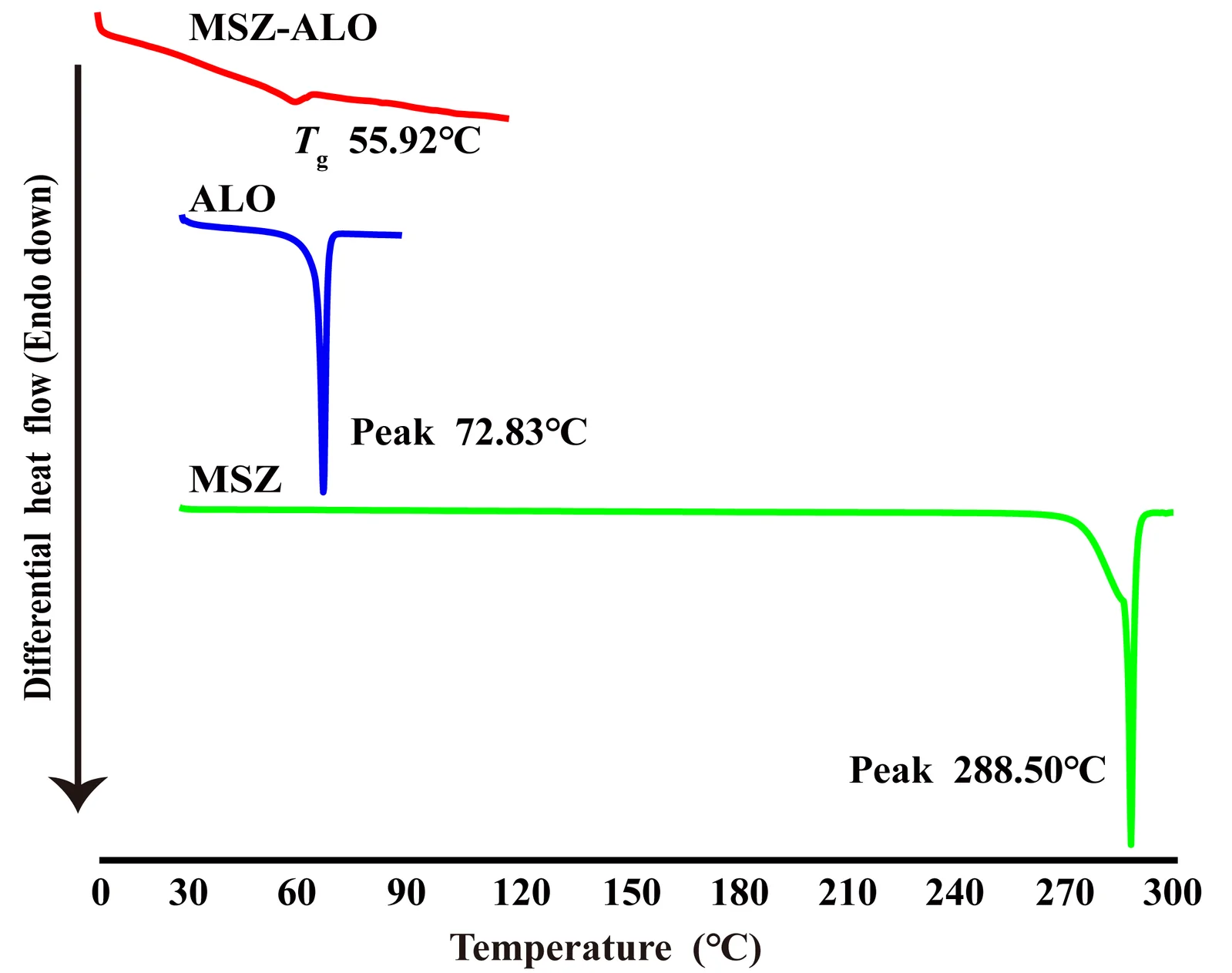
DSC curves of MSZ, ALO, and MSZ-ALO CAM.

To further confirm the CAM nature of the sample, DSC was employed. As shown in Figure 3, the DSC curves displayed a melting endotherm at 288.50 °C for MSZ and at 72.83 °C for ALO, consistent with reported values [24,36]. The measured *T*_fus_ and Δ*H*_fus_ values were 283.67 ± 2.83 °C and 634.76 ± 86.97 J/g for MSZ, and 70.65 ± 0.67 °C and 70.07 ± 2.30 J/g for ALO. The MSZ-ALO CAM exhibited only a single glass transition temperature (*T*_g_) at 55.92 °C, indicating the formation of a homogeneous mixed system [15]. The absence of sharp melting endotherms, together with the amorphous halo observed in PXRD, collectively confirmed that the system existed in an amorphous state [20].

### 3.3. FTIR Analysis

**Figure 4:**
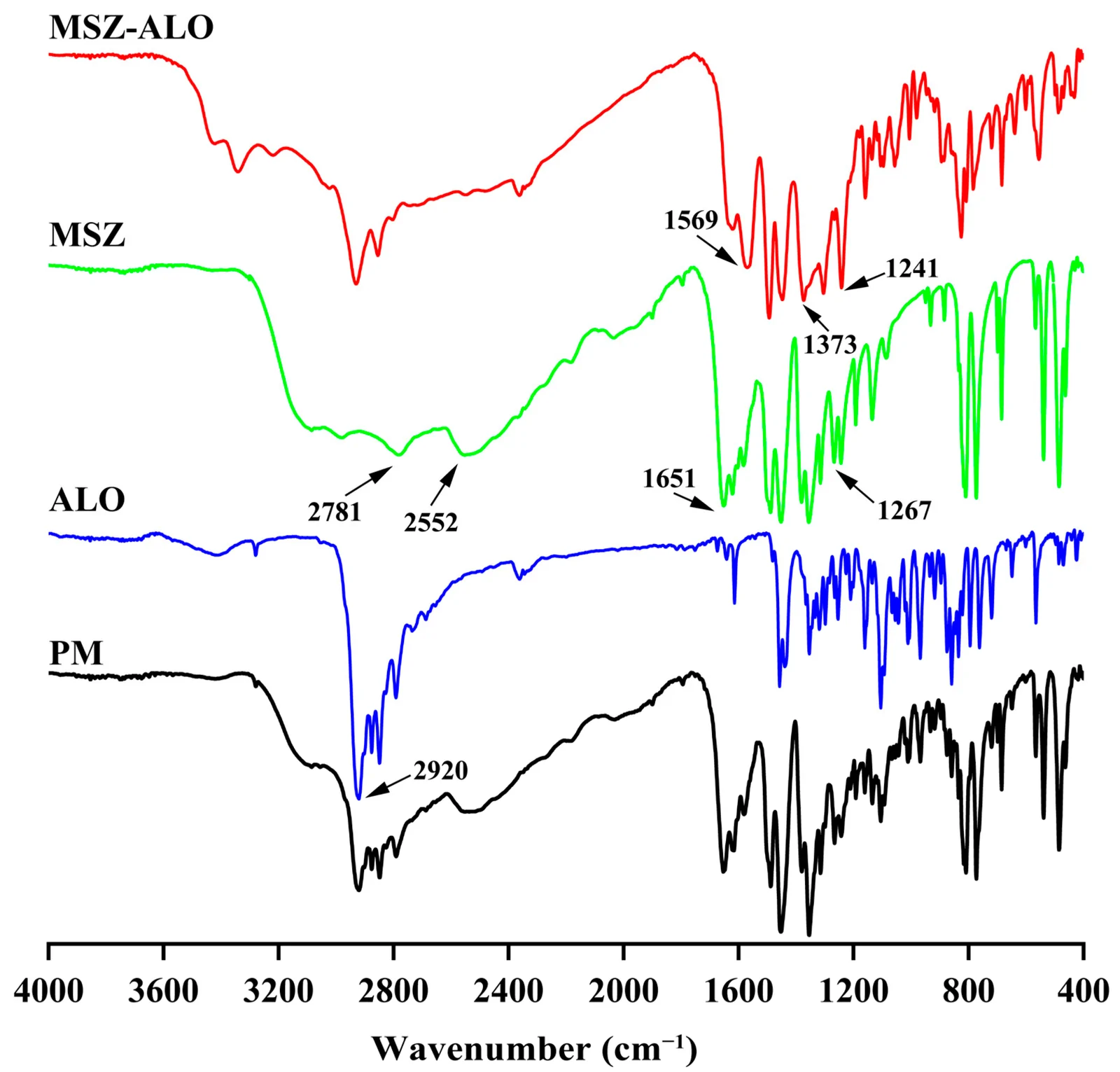
FTIR spectra of MSZ-ALO CAM, MSZ, ALO, and PM.

To elucidate the intermolecular interactions between MSZ and ALO in the CAM system, the FTIR spectra of MSZ, ALO, MSZ-ALO CAM, and PM were recorded, as shown in Figure 4. The spectrum of MSZ exhibited a hydrogen-bonded O–H stretching vibration of the carboxylic acid dimer in the range of 2000–3000 cm^−1^, a C=O stretching vibration at 1651 cm^−1^, an amino bending vibration at 1621 cm^−1^, a C–N stretching vibration at 1355 cm^−1^, and a phenolic C–O stretching vibration at 1267 cm^−1^, consistent with the literature [33,37,38,39]. ALO displayed an N–H stretching vibration at 2920 cm^−1^ and an N–H bending vibration in the range of 1640–1615 cm^−1^ [24]. The spectrum of PM merely exhibited a simple superposition of the characteristic peaks of the two individual components, indicating that simple physical mixing was insufficient to generate the intermolecular interactions observed in the CAM.

Compared with the spectra of the individual components, the IR spectrum of the MSZ-ALO CAM showed significant changes, suggesting molecular-level interactions and the formation of a new system. Specifically, the characteristic peak of the carboxylic acid dimer at 2552 cm^−1^ in neat MSZ completely disappeared, confirming the complete disruption of the original crystalline intermolecular hydrogen bonds. Furthermore, the C=O stretching vibration of the MSZ carboxyl group at 1651 cm^−1^ vanished, while two new absorption bands emerged at 1569 cm^−1^ and 1373 cm^−1^, corresponding to the asymmetric and symmetric stretching vibrations of the carboxylate group, respectively. These results indicated that a proton transfer may have occurred between the acidic carboxyl group of MSZ and the basic tertiary amine site of ALO, forming a carboxylate–ammonium ionic bond [24,40]. This interaction, which is stronger than intermolecular hydrogen bonding, serves as the dominant stabilizing force in the CAM system [19]. Moreover, the phenolic C–O stretching vibration of MSZ shifted from 1267 cm^−1^ to a lower wavenumber of 1241 cm^−1^, consistent with the spectroscopic rule for hydrogen bond formation: hydrogen bonding reduces the force constant, thereby lowering the vibrational frequency [41]. This indicated that the phenolic hydroxyl group of MSZ participated in intermolecular hydrogen bonding, with the protonated amine or secondary amine of ALO serving as the hydrogen acceptor, providing auxiliary stabilization to the system. Consequently, the CAM system was primarily stabilized by the ionic bond formed through proton transfer between the carboxyl group of MSZ and the amine group of ALO, complemented by hydrogen bonding involving the phenolic hydroxyl group.

### 3.4. ^1^H NMR Analysis

**Figure 5:**
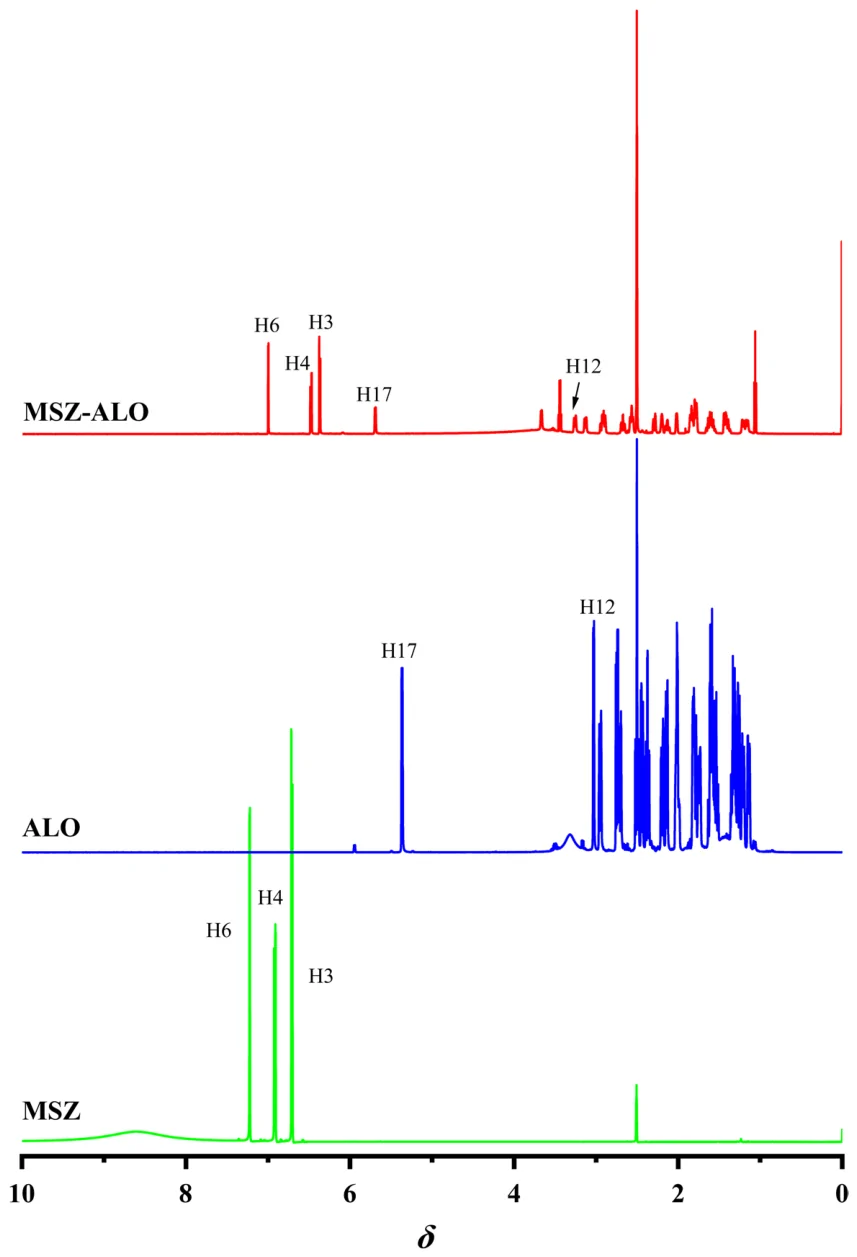
^1^H NMR spectra of MSZ-ALO CAM, ALO, and MSZ.

To elucidate the intermolecular interactions between MSZ and ALO in the CAM system, the ^1^H NMR spectra of MSZ, ALO, and MSZ-ALO CAM were recorded, as shown in Figure 5. The structures of MSZ and ALO were confirmed by comparison with the literature [42,43]. The spectrum of MSZ-ALO CAM retained the characteristic signals of both individual components while exhibiting clear chemical shift displacements, indicating that the two components were not simply physically mixed but rather formed an intermolecular interaction system. However, upon co-amorphization, all aromatic protons shifted upfield: the H6, H4, and H3 protons of MSZ moved from 7.22, 6.91, and 6.72 ppm to 7.00, 6.48, and 6.37 ppm, respectively. This upfield shift may be attributed to changes in the electron density on the benzene ring resulting from the participation of the carboxyl and phenolic hydroxyl groups of MSZ in ionic bonding and intermolecular hydrogen bonding. Furthermore, the H17 signal of ALO shifted downfield from 5.37 to 5.70 ppm, and the H12 signal shifted from 3.03 to 3.26 ppm. The simultaneous and pronounced downfield shifts in both the nitrogen α-hydrogens adjacent to the basic tertiary amine of ALO and the olefinic proton, contrasted with the uniform upfield shifts in the MSZ aromatic protons, provide mutual corroboration. In the ^1^H NMR spectrum of crystalline MSZ obtained in this study, a broad band was observed near *δ* 9 ppm. This signal was assigned to the exchangeable protons of the carboxyl group (–COOH), phenolic hydroxyl group (–OH), and aromatic amino group (–NH_2_). According to the literature, the intrinsic resonances of such labile protons typically appear near *δ* 5–6 ppm. However, these protons undergo pronounced downfield deshielding [43] owing to the combined effects of intramolecular hydrogen bonding, solute–solute and solute–solvent intermolecular interactions, and the electron-withdrawing conjugation effect of the aromatic carboxylic acid substituent. Rapid proton exchange further merges these signals into a single unresolved broad envelope. In the CAM sample, this broad signal disappeared, indicating deprotonation of the carboxylic acid and formation of a carboxylate–ammonium ion pair. Collectively, these results confirmed that proton transfer occurred from the carboxyl group to the tertiary amine nitrogen, generating an ionic bond that coexisted synergistically with hydrogen-bonding interactions. This molecular-level evidence demonstrated that the system was not a simple physical mixture but a CAM system successfully constructed through ionic interactions and hydrogen bonding. [44,45].

### 3.5. Accelerated and Long-Term Stability Assessment

Physical stability is a critical factor determining whether a CAM system can realize its solubility advantage and advance toward practical application. Amorphous systems are not constrained by lattice energy. They therefore exhibit markedly higher apparent solubility and dissolution rate than their crystalline counterparts [46]. This is the fundamental basis for improving the bioavailability of poorly water-soluble drugs. However, if physical stability is inadequate, the system may undergo recrystallization and phase transition during storage or dissolution. The drug then reverts to its thermodynamically stable crystalline form. Consequently, solubility drops sharply, and the previously gained dissolution advantage is instantly lost. This may even compromise the quality, stability, and efficacy of the final product [15,16].

**Figure 6:**
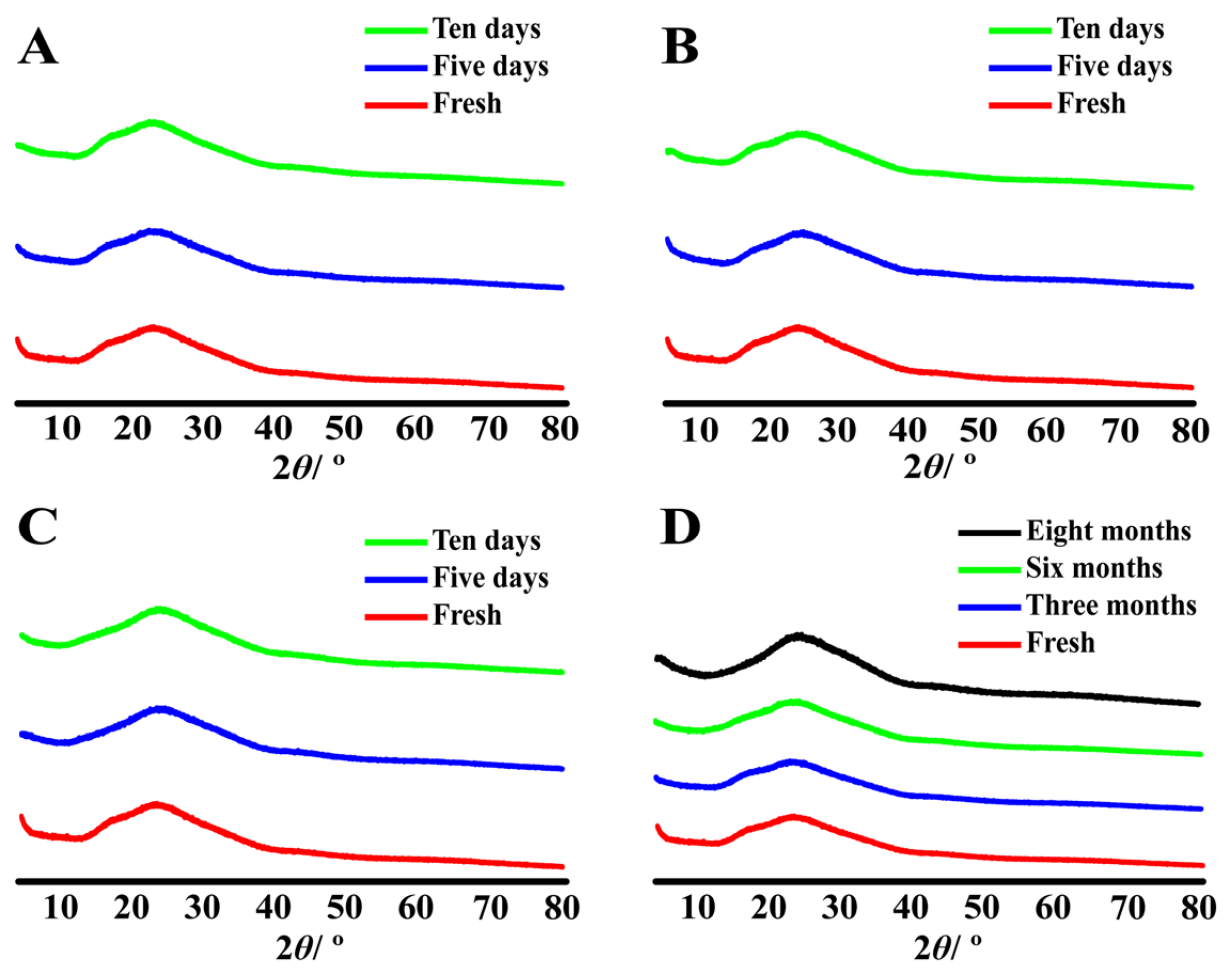
PXRD pattern of MSZ-ALO CAM in illumination (**A**), high temperature (at 50 °C) (**B**), high humidity (RH 75 ± 5%) (**C**), and dry conditions at 25 °C (**D**).

Accordingly, the MSZ-ALO CAM was subjected to accelerated and long-term stability studies. The PXRD patterns in Figure 6 show the following results. After 10 days of accelerated testing (50 °C, 75 ± 5% RH, 4500 ± 500 lx light) and 8 months of long-term storage (25 °C, dry environment), the MSZ-ALO CAM exhibited virtually no change. No crystalline diffraction peaks were observed, and no recrystallization occurred, indicating favorable physical stability. However, the glass transition temperature (*T*_g_) of MSZ-ALO CAM was only 55.92 °C. Prolonged storage above this temperature would transform the system from a glassy state to a rubbery state. In the glassy state, molecular motion is largely frozen. In the rubbery state, molecular mobility increases by orders of magnitude. This transition could trigger a series of physical and chemical changes, potentially compromising drug stability and functional efficacy. Therefore, storage in a dry environment at room temperature is recommended.

### 3.6. Solubility and Dissolution Rate Results

#### 3.6.1. Equilibrium Solubility Results

**Figure 7:**
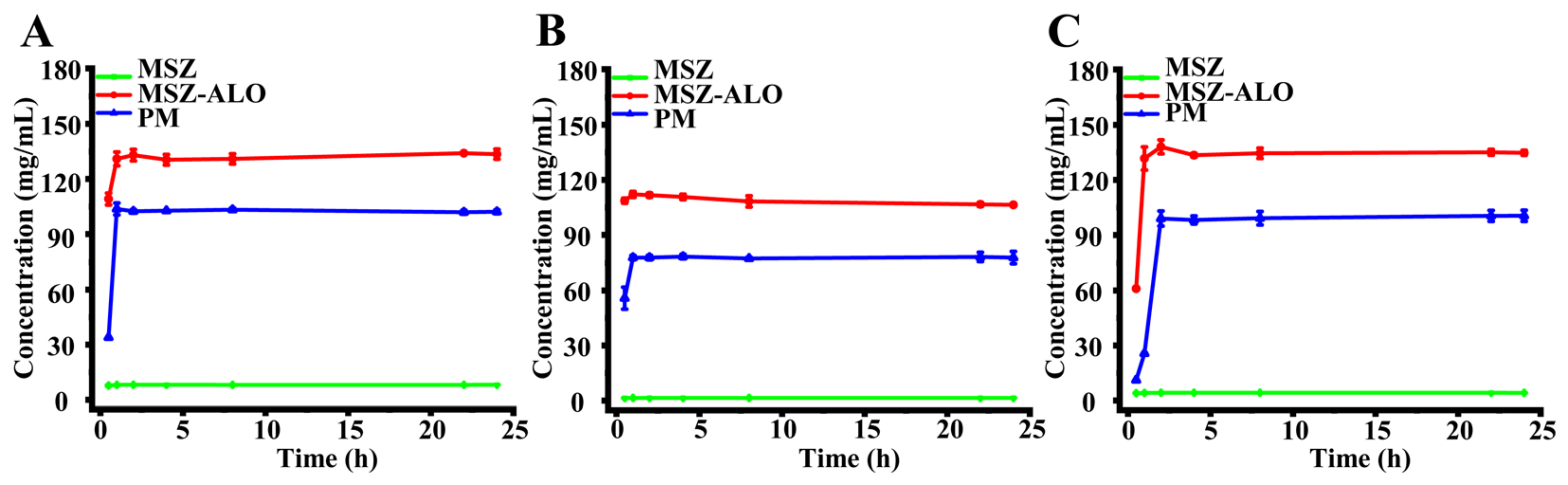
Equilibrium solubility of MSZ in pH 1.2 HCl solution (**A**), pH 4.5 acetate buffer (**B**), and pH 6.8 phosphate-buffered saline (**C**) at 37 ± 0.5 °C, using crystalline MSZ, MSZ-ALO CAM, or PM as the solid phase. The results are expressed as the mean ± standard deviation (SD) (n = 3).

The CAM systems can achieve higher equilibrium solubility than crystalline active pharmaceutical ingredients. The intermolecular interactions can inhibit recrystallization, allowing the supersaturated equilibrium state to be sustained over extended periods under non-sink conditions. By virtue of their high-energy state and hydrogen bonding and ionic interactions, CAM mixtures can persistently maintain elevated equilibrium concentrations [15]. Using crystalline MSZ, MSZ-ALO CAM, and PM as the solid phases, the equilibrium solubility of MSZ in various media was determined according to its concentration in the supernatant. As shown in Figure 7 and Table 1, the solubility of crystalline MSZ exhibited marked pH dependence, reaching its minimum at pH 4.5 (close to its isoelectric point) at only 1.48 ± 0.01 mg/mL, while the solubilities at pH 1.2 and pH 6.8 were 8.19 ± 4.89 mg/mL and 4.23 ± 0.04 mg/mL, respectively, consistent with previous reports [47]. Compared with crystalline MSZ, the MSZ-ALO CAM (as the solid phase) substantially enhanced the equilibrium solubility of MSZ in the dissolution medium across the entire pH range. The most pronounced solubilization was observed at pH 4.5, where the solubility was 71.9-fold higher than that of the crystalline drug, while 16.3-fold and 31.9-fold enhancements were achieved at pH 1.2 and pH 6.8, respectively. When PM was used as the solid phase, it also enhanced the equilibrium solubility of MSZ to a certain extent, but the solubilizing effect was weaker than that of the CAM system. It afforded 12.5-fold, 52.6-fold, and 23.8-fold improvements at pH 1.2, pH 4.5, and pH 6.8, respectively, relative to the crystalline drug. This indicated that the solubilization effect of the CAM system was not simply the sum of the two components but was intimately linked to the formation of the amorphous state and the accompanying intermolecular interactions. Furthermore, upon completion of the solubility test, the pH value of the pH 6.8 medium increased to 9.33. This pH drift is presumably attributable to the dissolution of the basic ALO component as well as the amphoteric character of MSZ. Nevertheless, PXRD analysis verified that the recovered solids retained the CAM characteristic without recrystallization, as demonstrated by the orange curve in Figure 2.

The solubilization effect of the MSZ-ALO CAM arises from the synergy between the high-energy amorphous state and intermolecular interactions [15]. Disordered molecular arrangement elevates Gibbs free energy and lowers the dissolution barrier [46], while hydrogen and ionic bonding between MSZ and ALO suppress MSZ aggregation and recrystallization post-dissolution, thereby sustaining high equilibrium concentrations. In contrast, the PM lacks such interactions, so MSZ recrystallizes more readily, and solubilization is weaker [44].

This pH-dependent behavior is particularly relevant for colonic delivery. MSZ is amphoteric, with carboxyl, phenolic hydroxyl, and amino groups that ionize with pH. Near its isoelectric point (pH ~4.5), net charge approaches zero and crystalline solubility is lowest [47,48]. In the CAM system, ALO, as an alkaloid, becomes protonated and positively charged in acidic to neutral media. It may form ion pairs with ionized MSZ, improving apparent hydrophilicity. Therefore, within the physiologically relevant pH range of the colon (approximately pH 5.5–7.5) [49], the combination of ion-pairing effects and the energetic advantage of the amorphous state is still sufficient to maintain enhanced apparent solubility.

Traditional strategies such as cyclodextrin complexation and polymer solid dispersions rely on inert excipients and face issues of low drug loading and safety risks [50,51]. Co-crystals have also shown limited improvement: a mesalazine–ascorbic acid co-crystal increased water solubility only 7-fold [3]. Bioactive ALO was employed as the co-former in the MSZ-ALO CAM system. The use of large amounts of polymeric excipients was avoided; the potential risk of excipient-related adverse effects was reduced, and MSZ solubility was enhanced by tens of folds, exceeding that of previously reported MSZ co-crystals. For UC, incomplete dissolution of crystalline MSZ in the colon leaves substantial solid drug unabsorbed, resulting in insufficient local exposure [52]. Highly-soluble CAM systems enable rapid and complete dissolution in simulated intestinal media and rapidly elevate free-drug concentration, which theoretically permits efficient local drug exposure [20]. The mucin network of mucus can trap solid particles via size-exclusion and adsorption effects, whereas dissolved small-molecule drugs exhibit less diffusion hindrance across mucus barriers [53]. In UC lesions, notwithstanding disruption of the compact inner mucus layer, the accumulation of loose outer mucus at inflamed sites further promotes solid-particle retention [54], an effect that may be partially mitigated by the dissolved-state advantage of the CAM. Therefore, the MSZ-ALO CAM may address the issue of incomplete colonic dissolution and improve local drug exposure, thereby supporting the further development of local colonic drug delivery systems based on this CAM.

#### 3.6.2. IDR Results

**Figure 8:**
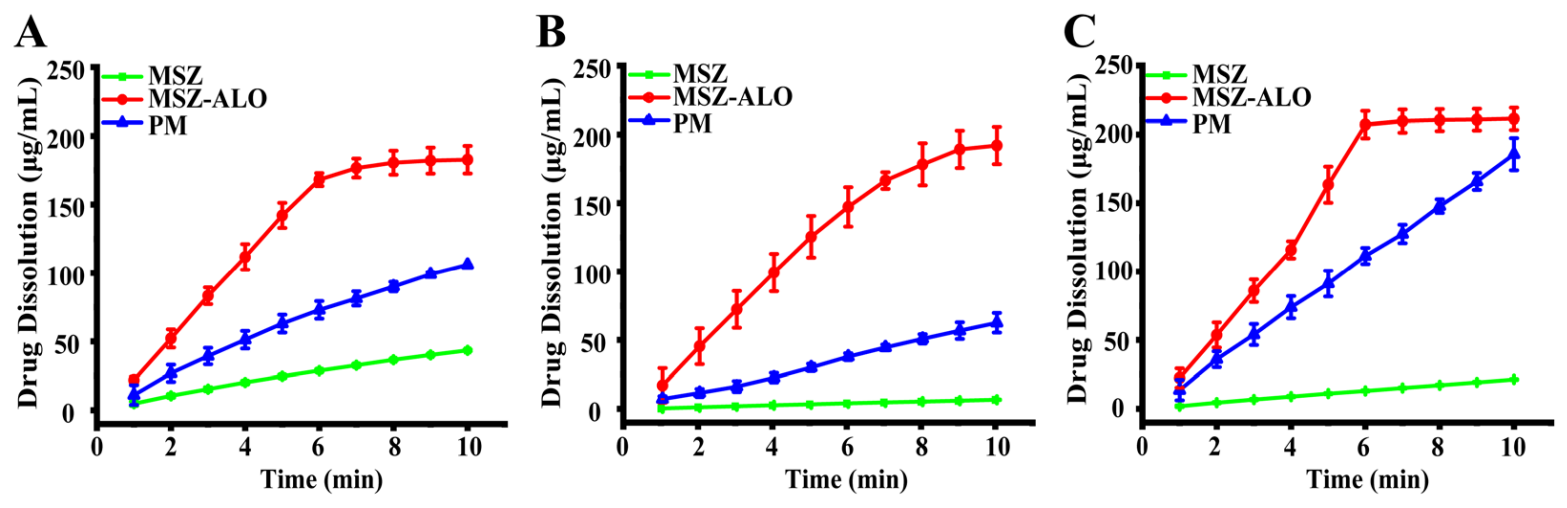
IDR patterns of MSZ in pH 1.2 HCl solution (**A**), pH 4.5 acetate buffer (**B**), and pH 6.8 phosphate-buffered saline (**C**) at 37 ± 0.5 °C, using crystalline MSZ, MSZ-ALO CAM, or PM as the compacted solid phase. The results are expressed as the mean ± standard deviation (SD) (n = 3).

To evaluate the dissolution characteristics of the CAM system, the IDR of MSZ was determined in different media, using crystalline MSZ, MSZ-ALO CAM, or PM as the compacted solid phase. As shown in Figure 8 and Table 2, the overall trend was highly consistent with the equilibrium solubility results. For crystalline MSZ, the IDR was entirely governed by its amphoteric ionization properties. At pH 4.5, near the isoelectric point, MSZ is electrically neutral and exhibits a stable crystal lattice and weak hydration. Consequently, the kinetic dissolution resistance is maximized, yielding the lowest IDR of 155.64 ± 0.73 μg/(min·cm^2^). Deviation from the isoelectric point, whether via protonation at pH 1.2 or deprotonation at pH 6.8, weakens the crystal lattice. The IDR thereby increases to 1011.07 ± 17.69 and 469.65 ± 14.72 μg/(min·cm^2^), respectively [55]. For the PM, MSZ exhibited a higher IDR than crystalline MSZ under all the tested pH conditions. However, the improvement was limited. This indicates that simple physical mixing cannot disrupt the ordered crystal lattice of MSZ.

In contrast, when MSZ-ALO CAM was used as the solid phase, the IDR of MSZ increased substantially in all test media. In pH 4.5 medium, the IDR of MSZ from this CAM was approximately 35.8-fold that from crystalline MSZ. At pH 1.2 and pH 6.8, the IDR of MSZ from the CAM increased by 6.2-fold and 16.6-fold, respectively. This was fully consistent with the trends observed in the equilibrium solubility. Notably, the 16.6-fold IDR enhancement at pH 6.8 is particularly relevant to colonic delivery, as it indicates that the CAM confers a marked kinetic dissolution advantage within the pH range typical of the colon. Overall, the CAM strategy was most advantageous near the isoelectric point of MSZ, where crystalline MSZ showed the most severe dissolution bottleneck, and still provided considerable enhancement under pH conditions relevant to the colon.

### 3.7. Permeability Results

MSZ is a BCS Class IV drug with low solubility and low permeability. Colonic pH varies among individuals and along different colonic segments. Normal pH ranges from 5.26 to 6.72 in the proximal colon and is approximately 6.6 in the distal colon [56]. In UC, pH decreases mainly during active disease, whereas pH in remission is near normal or only slightly reduced [57]. We therefore chose pH 5.0 and pH 6.5 to simulate the intestinal buffer media of active and remission UC, respectively. Passive paracellular transport participates in the intestinal transepithelial transport of MSZ [58]. We used the parallel artificial membrane permeability assay (PAMPA) to measure flux (*J*) and effective permeability (*P*_e_) of MSZ in vitro. Results are shown in Table 3. All samples were dissolved in DMSO, which eliminated solubility limitations. At pH 5.0, both *J* and *P*_e_ of MSZ were significantly higher than at pH 6.5 for all three formulations. This difference arises because at pH 6.5 most MSZ is deprotonated to an anionic form, which reduces membrane partitioning. At pH 5.0, near the isoelectric point, the neutral species predominates and increases lipophilicity, thus providing a stronger driving force for transmembrane diffusion [55,59]. At pH 5.0, *J* was comparable among crystalline MSZ, CAM, and PM, whereas *P*_e_ for CAM and PM was approximately 1.3-fold higher than that for crystalline MSZ. At pH 6.5, both *J* and *P*_e_ for CAM and PM were approximately twice those of crystalline MSZ, and the two formulations exhibited comparable performance.

For BCS IV drugs, formulations with higher solubility provide a stronger concentration driving force. This helps overcome the barrier of low permeability [60]. At pH 5.0, which mimics active UC, MSZ is near its isoelectric point and exists mainly as neutral molecules. Combined with the results of equilibrium solubility and IDR, the MSZ-ALO CAM exhibits higher solubility and dissolution rate than crystalline MSZ. It provides more dissolved drug locally in the colon, and its effective permeability is therefore increased. At pH 6.5, simulating remission or a normal colonic environment, MSZ exists mainly as anions, which reduces membrane permeation. In the CAM, however, ALO forms ion pairs with MSZ, neutralizing its negative charge and alleviating membrane repulsion, which markedly improves the transmembrane transport of the anionic drug; such pH-related enhancement is most pronounced under weakly alkaline conditions where the drug is fully ionized [61], raising permeability approximately 2-fold. Meanwhile, the CAM itself exhibits relatively high solubility and a rapid dissolution rate, which may help sustain an adequate concentration of dissolved drug in the colon, thereby partially compensating for the intrinsically low permeability of MSZ and favoring its accumulation in the local mucosa.

In summary, because crystalline MSZ delivers only limited dissolved drug to the colon under both pH conditions, its anti-inflammatory efficacy may be restricted [48]. The PM can improve permeability with the help of ALO, but it lacks stable intermolecular interactions. Dissolved MSZ in PM thus faces a recrystallization risk [17], which may compromise the solubility advantage over time. By contrast, the CAM exhibits high solubility, rapid dissolution, and enhanced permeability, properties that may help maintain dissolved drug concentrations and transmembrane efficiency at the colonic site, providing a basis for the further development of local delivery strategies for UC therapy.

### 3.8. Network Pharmacology Analysis of the Synergistic Anti-Ulcerative Colitis Mechanism

Network pharmacology analyzes drug actions at the systems level via multi-target and multi-pathway mechanisms. It is now widely applied in mechanism discovery. Previous network pharmacology studies have systematically elucidated the potential targets and signaling pathways of MSZ in UC treatment. They identified core targets such as TNF, IL1B, PTGS2, and EGFR. MSZ primarily acts by modulating NF-κB-mediated inflammation and maintaining intestinal immune homeostasis [62]. For ALO, a dedicated network pharmacology study on UC intervention is lacking. Nevertheless, in vivo and in vitro experiments have shown that ALO can ameliorate experimental colitis. It does so by regulating PI3K/Akt/mTOR, MAPK, and NF-κB pathways [26,63]. Network pharmacology offers advantages in deciphering compound mechanisms through a multi-component, multi-target, and multi-pathway approach. Analyzing a single drug alone cannot capture the integrated effects of combination therapy. MSZ has anti-inflammatory and immunomodulatory activities. It may synergize with the anti-inflammatory action of ALO. Together, they can target multiple pathological mechanisms of UC. The MSZ-ALO CAM formulation is therefore expected to exert multi-target therapeutic effects as a single agent.

**Figure 9:**
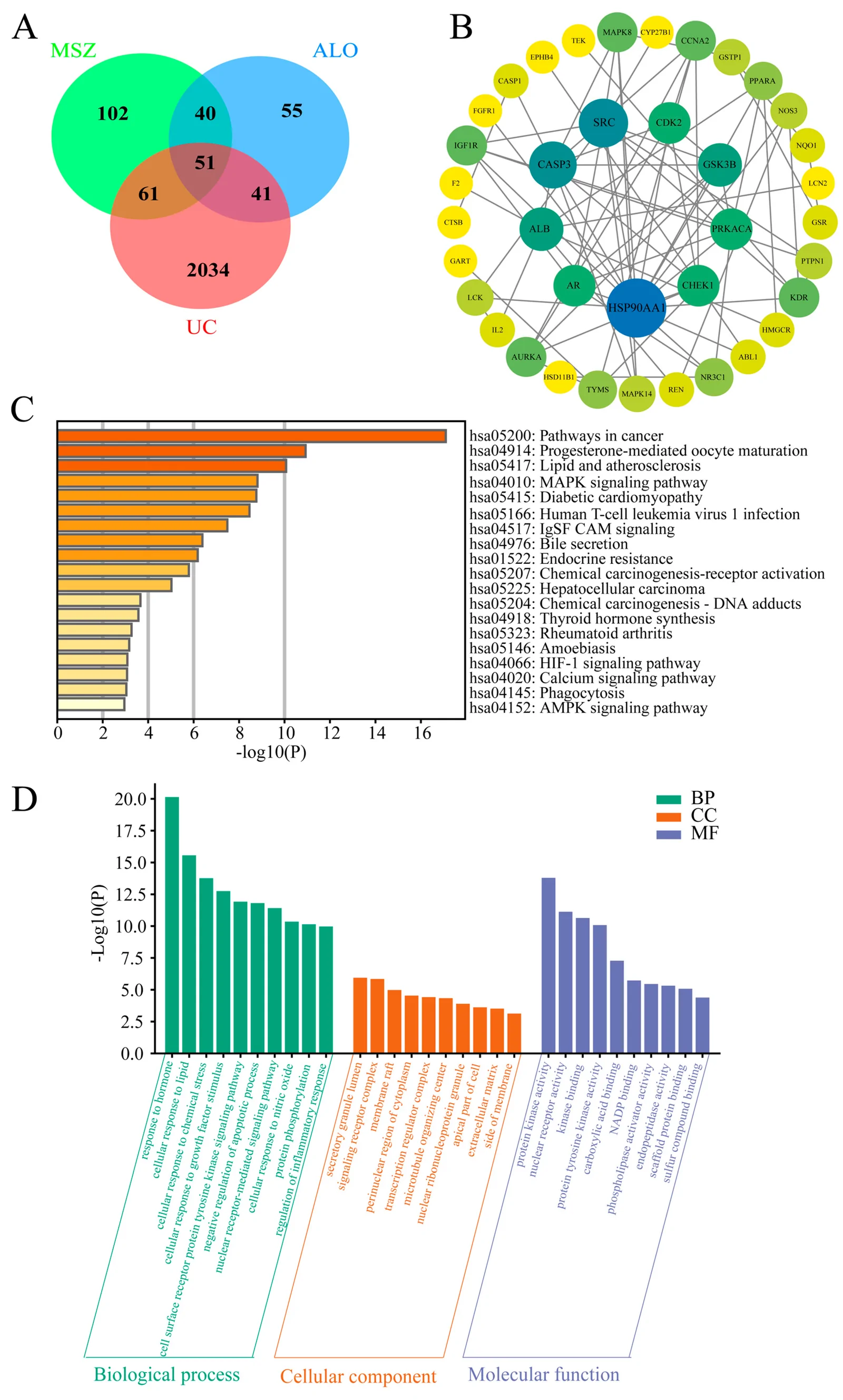
Network pharmacology analysis of MSZ-ALO CAM. Venn diagram of targets of MSZ, ALO, and UC (**A**); the PPI network of intersection targets of UC with MSZ and ALO (**B**); KEGG enrichment analysis of potential targets (top 19) (**C**); GO enrichment analysis of potential targets (top 10) (**D**).

To elucidate the synergy between MSZ and ALO, the common target sets of both drugs were integrated. The overlap with UC disease targets was then focused on. A synergistic network pharmacology analysis was subsequently performed. In this study, SwissTargetPrediction predicted 13 potential targets for MSZ and 40 for ALO, while PharmMapper predicted 248 potential targets for MSZ and 151 for ALO. After merging and removing duplicates, 254 potential targets were obtained for MSZ, and 187 for ALO. The OMIM database yielded 104 UC-related targets, and the GeneCards database, with a relevance score threshold set at or above the median, yielded 2173 UC-related targets; merging and deduplication resulted in a total of 2187 UC-related targets. The Venn diagram (Figure 9A) revealed 51 common targets shared among the three sets, providing molecular-level evidence for a synergistic target basis upon combining ALO with MSZ. Further analysis of the protein–protein interaction (PPI) network (Figure 9B), with the minimum required interaction score set at 0.700, identified the top five hub targets as heat shock protein 90 alpha family class A member 1 (HSP90AA1), non-receptor tyrosine-protein kinase (SRC), caspase-3 (CASP3), glycogen synthase kinase-3 beta (GSK3B), and albumin (ALB). KEGG pathway analysis using Metascape (Figure 9C) revealed enrichment in the MAPK signaling pathway, HIF-1 signaling pathway, and AMPK signaling pathway. GO analysis (Figure 9D) encompassing biological process (BP), cellular component (CC), and molecular function (MF) categories showed that the differentially enriched genes were mainly involved in biological processes such as cellular responses to hormones/lipids/growth factors, protein phosphorylation, negative regulation of apoptosis, and inflammatory responses; the enriched cellular components were primarily membrane rafts, membrane receptor complexes, the perinuclear region, the extracellular matrix, and secretory granules; the enriched molecular functions were dominated by protein kinase activity, tyrosine kinase activity, nuclear receptor activity, and small-molecule binding.

Among these hub targets, HSP90AA1, as a critical molecular chaperone, stabilizes multiple pro-inflammatory signaling proteins, and its inhibition has been demonstrated to alleviate colonic inflammation in animal models [64,65]. SRC family kinases are aberrantly overexpressed and activated in the colonic mucosa of UC patients and are considered to be closely associated with impaired intestinal epithelial barrier function and the secretion of pro-inflammatory cytokines [66]. In UC, CASP3, as the terminal executioner of apoptosis, is excessively activated by upstream signals such as ROS and ER stress, directly leading to intestinal epithelial cell death and loss of barrier function, thus representing a key node in maintaining mucosal barrier integrity [67]. GSK3B is an upstream kinase that regulates the activity of pro-inflammatory pathways such as NF-κB, and selective inhibition of GSK3B activity can effectively attenuate the inflammatory response in experimental colitis [68]. Serum ALB not only serves as a classic clinical indicator reflecting UC activity and nutritional status but also plays a direct role in maintaining colloid osmotic pressure and antioxidant defense [69]. Collectively, these hub targets span multiple levels ranging from apoptosis regulation and barrier maintenance to inflammatory signal transduction, revealing the possible mechanisms underlying the synergistic therapeutic effects of these two drugs against UC.

Further KEGG pathway enrichment analysis indicated that these common targets were predominantly enriched in the MAPK signaling pathway, HIF-1 signaling pathway, and AMPK signaling pathway. In the pathogenesis of UC, aberrantly activated MAPK pathways amplify intestinal inflammation, persistently accumulated HIF-1α mediates hypoxic injury and lesion progression, and the inactivation of AMPK signaling compromises barrier protection and metabolic homeostasis. The interplay among these three pathways jointly drives mucosal inflammation, barrier disruption, and ulcer formation. The MAPK pathway is a core signaling cascade that drives the massive synthesis of pro-inflammatory cytokines in UC. In the setting of pronounced inflammatory infiltration in the colonic mucosa, LPS induces excessive phosphorylation of p38, JNK, and ERK in epithelial cells and macrophages, activating NF-κB and releasing large quantities of pro-inflammatory factors, while also degrading ZO-1 and occludin, thereby disrupting the intestinal barrier and aggravating mucosal erosion and apoptosis [70]. MSZ has been reported to inhibit the phosphorylation and activation of key kinases in the MAPK pathway [71,72,73], and ALO can also exert anti-inflammatory effects by suppressing p38/JNK/ERK activation [74]. The HIF-1 pathway is significantly activated in the inflammatory hypoxic microenvironment of UC [75]. Reduced blood flow at inflammatory sites and a surge in oxygen consumption by immune cells create pathological mucosal hypoxia, leading to substantial HIF-1α accumulation. While short-term mild stabilization of HIF-1α can promote barrier repair, persistently high levels of HIF-1α promote the release of pro-inflammatory cytokines, aberrant angiogenesis, intestinal fibrosis, and an elevated risk of colitis-associated carcinogenesis [76]. The hub target HSP90AA1 identified in this study is a key molecular chaperone that stabilizes the protein conformation of HIF-1α, suggesting a potential interventional route that may indirectly modulate HIF-1 signaling through regulation of HSP90AA1 [77]. The AMPK pathway, serving as a central regulator of cellular energy metabolism, can both directly inhibit the transcriptional activity of NF-κB and phosphorylate GSK3B to render it inactive, thereby forming a negative feedback loop that limits the amplification of pro-inflammatory signals [78]. Therefore, these three pathways are of considerable significance for the synergistic treatment of UC with MSZ and ALO.

In summary, this study postulates that MSZ and ALO may act through core nodes such as HSP90AA1 and SRC, coordinately modulating the MAPK, HIF-1, and AMPK signaling pathways to exert combined therapeutic effects on UC via multiple mechanisms, including inhibition of epithelial cell apoptosis, protection of the mucosal barrier, and blockade of the stepwise amplification of inflammatory signals. The pharmacological network analysis performed herein serves as an early-stage in silico theoretical screening. This analysis aims to preliminarily evaluate whether the MSZ-ALO CAM system fulfills our dual design rationale: improved solubility for optimized pharmacokinetic profiles and potential synergistic anti-inflammatory pharmacodynamic effects between MSZ and ALO. It should be emphasized that in silico predictions carry intrinsic limitations and cannot provide definitive biological evidence. To confirm whether the MSZ-ALO CAM can realize the aforementioned synergistic effects, systematic in vitro-to-in vivo validation is required in subsequent investigations.

## 4. Conclusions

In this study, an MSZ-ALO CAM system was constructed for the first time. It is the first CAM formulation combining MSZ with a small-molecule natural product derived from traditional Chinese medicine. The system markedly enhanced the solubility of MSZ. At pH 4.5, the equilibrium solubility was 71.9-fold higher than that of crystalline MSZ. This enhancement is substantially greater than that of previously reported MSZ co-crystals. The IDR was also improved by up to 35.8-fold. In addition, the permeability at pH 6.5 was approximately doubled. The system exhibited excellent physical stability under long-term and accelerated conditions, with no recrystallization observed. Network pharmacology identified 51 common targets shared by MSZ, ALO, and UC. These targets were enriched in the MAPK, HIF-1, and AMPK signaling pathways. Core hub targets included HSP90AA1, SRC, CASP3, GSK3B, and ALB. This multi-target profile suggests potential for synergistic treatment of UC. Collectively, the CAM system of MSZ-ALO combines high solubility, rapid dissolution, improved permeability under colonic pH conditions, and favorable physical stability, which provides both formulation and mechanistic foundations for local colonic delivery of MSZ. Further in vivo pharmacokinetic and pharmacodynamic investigations are warranted to validate its therapeutic potential.

## Figures and Tables

**Table 1:** Equilibrium solubility of MSZ in various media when the solid phase was crystalline MSZ, MSZ-ALO CAM, or PM. The results are expressed as the mean ± standard deviation (SD) (n = 3).

Dissolution Media	MSZ (mg/mL)	MSZ-ALO CAM (mg/mL)	PM (mg/mL)
pH 1.2	8.19 ± 4.89	133.53 ± 2.75	102.15 ± 1.08
pH 4.5	1.48 ± 0.01	106.39 ± 0.85	77.92 ± 3.35
pH 6.8	4.23 ± 0.04	134.84 ± 1.36	100.49 ± 3.04

**Table 2:** IDR of MSZ in different media using crystalline MSZ, MSZ-ALO CAM, or PM as the compacted solid phase. The results are expressed as the mean ± standard deviation (SD) (n = 3).

Dissolution Media	MSZ [μg/(min·cm^2^)]	MSZ-ALO CAM [μg/(min·cm^2^)]	PM [μg/(min·cm^2^)]
pH 1.2	1011.07 ± 17.69	6244.70 ± 280.21	2604.10 ± 48.34
pH 4.5	155.64 ± 0.73	5568.25 ± 265.37	1302.48 ± 14.44
pH 6.8	469.65 ± 14.72	7778.85 ± 290.18	4087.98 ± 59.63

**Table 3:** Permeation flux (*J*) and effective permeability (*P*_e_) of MSZ in different media using crystalline MSZ, MSZ-ALO CAM, or PM as the donor phase. The results are expressed as the mean ± standard deviation (SD) (n = 3).

Groups	pH 5.0		pH 6.5	
	*J* [μg/(min·cm^2^)]	*P*_e_ (cm/s)	*J* [μg/(min·cm^2^)]	*P*_e_ (cm/s)
MSZ	0.0117 ± 4.78 × 10^−4^	5.77 × 10^−7^ ± 1.60 × 10^−7^	0.00163 ± 1.40 × 10^−5^	7.43 × 10^−8^ ± 3.62 × 10^−9^
MSZ-ALO	0.0111 ± 5.01 × 10^−4^	7.52 × 10^−7^ ± 4.07 × 10^−7^	0.00328 ± 1.56 × 10^−4^	1.58 × 10^−7^ ± 4.42 × 10^−9^
PM	0.0113 ± 4.38 × 10^−4^	7.16 × 10^−7^ ± 2.83 × 10^−7^	0.00359 ± 5.28 × 10^−5^	1.69 × 10^−7^ ± 3.98 × 10^−9^

## Data Availability

The data presented in this study are available on request from the corresponding author.

## Abbreviations

The following abbreviations are used in this manuscript:

ALO	Aloperine
CAM	Co-amorphous
DSC	Differential Scanning Calorimetry
FTIR	Fourier Transform Infrared Spectroscopy
HPLC	High-performance liquid chromatography
IDR	Intrinsic Dissolution Rate
MSZ	Mesalazine
NMR	Nuclear Magnetic Resonance
PM	Physical mixture
PXRD	Powder X-ray Diffraction
SD	Standard deviation
UC	Ulcerative colitis
